# Changing Perspectives from Oxidative Stress to Redox Signaling—Extracellular Redox Control in Translational Medicine

**DOI:** 10.3390/antiox11061181

**Published:** 2022-06-16

**Authors:** Paola Loreto Palacio, José R. Godoy, Orhan Aktas, Eva-Maria Hanschmann

**Affiliations:** 1 Department of Neurology, Medical Faculty, Heinrich Heine University, Moorenstraße 5, 40225 Düsseldorf, Germany; paolaloreto1703@gmail.com (P.L.P.); orhan.aktas@med.uni-duesseldorf.de (O.A.); 2 Department of Veterinary Biomedical Sciences, Long Island University College of Veterinary Medicine, 720 Northern Boulevard, Brookville, NY 11548, USA; jose.godoy@liu.edu

**Keywords:** oxidative stress, extracellular redox regulation, reactive oxygen species, glutathione, thioredoxin proteins, translational medicine, biomarkers

## Abstract

Extensive research has changed the understanding of oxidative stress that has been linked to every major disease. Today we distinguish oxidative eu- and distress, acknowledging that redox modifications are crucial for signal transduction in the form of specific thiol switches. Long underestimated, reactive species and redox proteins of the Thioredoxin (Trx) family are indeed essential for physiological processes. Moreover, extracellular redox proteins, low molecular weight thiols and thiol switches affect signal transduction and cell–cell communication. Here, we highlight the impact of extracellular redox regulation for health, intermediate pathophenotypes and disease. Of note, recent advances allow the analysis of redox changes in body fluids without using invasive and expensive techniques. With this new knowledge in redox biochemistry, translational strategies can lead to innovative new preventive and diagnostic tools and treatments in life sciences and medicine.

## 1. Changing Perspectives—From Oxidative Stress to Redox Signaling

Oxidative stress has been linked to basically every major disease. Excessive levels of reactive oxygen species (ROS) are known to be produced by exogenous and endogenous sources and eventually lead to irreversible modifications of biomolecules and damage of biological structures and tissues. Therapies administering radical-scavenging antioxidants seemed like an obvious therapeutic approach to counteract conditions of oxidative stress. Even though they have been effective in cellular and animal models, antioxidant supplements have mostly failed in clinical studies [1]. Explanations include low dosages, ultra-fast reaction rates and short study durations as well as limitations due to antioxidant selection or potential pro-oxidant effects [2,3]. Since antioxidants scavenge free ROS they encounter, the reducing power within a system can be increased, which leads to so-called reductive stress—a term first mentioned by Albert Wendel [4]. Reductive stress has also been linked to different pathologies and describes an abnormal increase of reducing equivalents (e.g., GSH, NADPH) and activation of antioxidant systems [1,5]. Originally, all these findings have led to the assumption that oxidants and reductants need to be balanced. However, what we have learned from extensive research ever since is that specific reactive species are in fact essential for signal transduction and physiological processes and therefore indispensable for life. Therefore, antioxidant treatments most likely interfere with physiological signaling and their use in clinical practice should be tailored to the pathology to prevent undesired effects.

**Figure 1:**
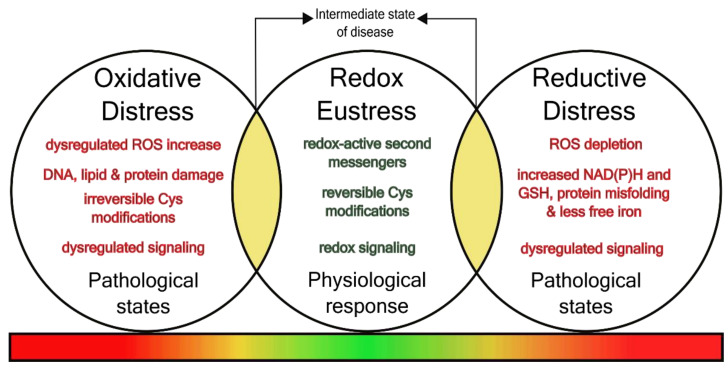
Redox signaling states in physiology and pathology. Under physiological conditions (redox eustress), redox-active second messengers such as O2, NO act on thiol switches of target proteins inducing reversible cysteine modifications and activation of redox signaling. Dysregulated redox signaling caused, either by ROS increase or depletion, leads to oxidative or reductive stress, respectively. The transition from physiological to a pathological state represents a diagnostically relevant, but hitherto not well characterized “intermediate state” (yellow areas).

The commonly used balance constituted an outdated illustration for oxidative stress. In fact, a new definition was introduced that acknowledges new findings on (i) the absence of a general redox equilibrium, (ii) enzymatic production and decay of reactive oxygen species, (iii) the presence of regulatory thiol switches of proteins, and (iv) the physiological implications of redox-mediated signal transduction. The new definition describes oxidative stress as a general disruption of physiological redox signaling and redox regulation and was introduced by Dean Jones in 2006 [6]. Indeed, Helmut Sies, who introduced the term oxidative stress 35 years ago, summarized the merits and pitfalls of this term and recently expanded this new definition by subdividing oxidative stress into oxidative eustress and distress. Eustress describes the physiological implications of specific second messengers like hydrogen peroxide (H_2_O_2_) and oxidative posttranslational modification of proteins in signal transduction and redox control. Oxidative distress, on the other hand, describes the excessive and uncontrolled oxidant challenge that disrupts physiological signaling and induces damage and disease [7] (Figure 1). Of note, these definitions are also applicable to nitrosative stress. The second messenger nitric oxide (NO) has also been shown to function in specific redox-mediated signaling via thiol nitrosylation and has clinical implications in many disorders. The overall significance and impact of redox regulation becomes evident, when we look at the human proteome that contains 214,000 cysteines (Cys). Depending on cell type and tissue, 5–12% of the total protein Cys are oxidized [8]. It has been predicted that almost every signaling cascade contains at least one redox-sensitive element, linking redox metabolism inevitably to cellular and biological function [8].

**Figure 2:**
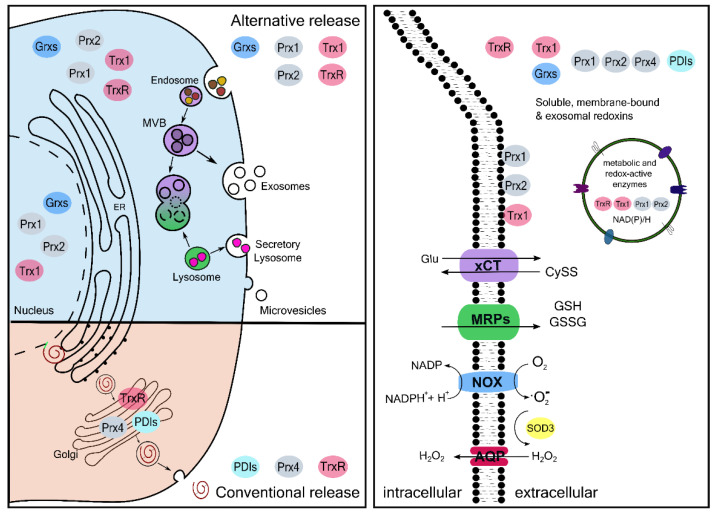
Intracellular and extracellular distribution of redoxins. Subcellular localization, presence of the redoxins in the extracellular and intravascular space, as well as in extracellular vesicles. Unconventional secretion pathways of protein: the majority of the redoxins are secreted via these pathways; some of them are Trx, TrxR, Prx1, Prx2. Conventional secretion pathway: from the thioredoxin family protein, PDIs and Prx4 are known to be secreted via the conventional pathway, possessing a leader peptide that enables their secretion. Representation of ROS generation as part of the redox signaling. TLR4: Toll-like receptor 4. GSH: Glutathione. NOX: NADPH oxidase. AQP: Aquaporin. SOD: Superoxide dismutase. Prx: Peroxiredoxin. Trx: Thioredoxin. TrxR: Thioredoxin reductase. EV: Extracellular vesicle.

Thiol switches have been described in many proteins functioning in different cellular pathways. Interestingly, not all thiol switches function as on–off switches. In fact, they can be allosteric switches that modulate enzymatic activity, they can regulate protein distribution and interactions and one specific Cys residue can be altered by different modifications affecting its role in several pathways [8]. Oxidative modifications include for instance the formation of sulfenic acids, inter- or intramolecular disulfide bridges, S-nitrosylation, S-sulfhydration or S-glutathionylation by the formation of a mixed-disulfide with glutathione. All of these modifications are rapid, specific and reversible. As such, they are enzymatically controlled by oxidoreductases of the Thioredoxin (Trx) family. We are only beginning to understand how these modifications affect protein conformation, translocation, and function and additionally how they regulate the interplay with other posttranslational modifications and interaction with ligands, proteins and potential therapeutics. Most of the research in the field of redox biology has focused on the intracellular role of redox regulation, even though redox regulation also occurs in the extracellular space. Reactive species can cross membranes (e.g., hydrogen peroxide via aquaporins) and can even be produced at the plasma membrane. Interestingly, these enzymes show a distinct expression pattern in polarized cells (PolarProtDb, [9]): aquaporin 3 is expressed on the basolateral membrane; aquaporin 8 and 9, as well as the oxidases Duox1 and 2, are expressed on the apical membrane. In addition, small molecular weight thiols, redoxins and antioxidants (e.g., superoxide dismutase 3, glutathione peroxidases) are present in the extracellular space. They are specifically secreted via classical and non-classical pathways for instance in extracellular vesicles upon distinct stimuli (Figure 2) [10,11].

Redox proteins were shown to be able to bind to components of the extracellular matrix or the extracellular surface of the plasma membrane. Even though the extracellular functions are not well documented, oxidative modifications and regulatory thiol switches have been described in soluble proteins and ectodomains of membrane proteins (recently reviewed in [12]). Thus, cells can modulate their extracellular redox environment and affect autocrine, endocrine and paracrine signaling.

Compared to the intracellular space, the extracellular fluid is a more oxidizing environment. The major redox couples found in human plasma are Cys/CySS and GSH/GSSG, being the former more oxidized (−80 mV) than the latter (−140 mV) [13]. Concentrations of cysteine/cystine and GSH/GSSG are comparably lower. The extracellular levels of glutathione, for instance, range between 2 and 20 µM [14]. However, these concentrations are mostly measured in total body fluids. The concentrations in close proximity to distinct cell types might be significantly higher. H_2_O_2_ is the most relevant (non-radical) reactive species in the extracellular space, possesses a relatively long half-life and is able to diffuse out of cells and travel with the extracellular fluid, affecting targets that are more distant [15]. In addition to this, H_2_O_2_ levels in the blood plasma are 100-fold higher than those inside cells under physiological conditions [16]. Thus, it is a main component in extracellular redox signaling [7]. Extracellularly located enzymes such as Gpxs and Prxs catalyze the reduction of H_2_O_2_ [17]. As specific cell types are equipped with different sets and amounts of redox active proteins and molecules, so are different types of body fluids. The lining fluid of the lung, for instance, contains high levels of reduced GSH, released by epithelial cells [18].

Extracellular GSH can, for instance, scavenge and detoxify hypochlorous acid (HOCl) [19]. Interestingly, it has been shown that astrocytes release GSH to provide GSH and Cys for neurons [20]. This depends on neuronal activity and Nrf-2 dependent induction of genes linked to glutathione metabolism in astrocytes [21]. Thus, extracellular and transcellular redox control is important for signal transduction and cell–cell communication, and potentially systemic reactions. Particularly, the role of extracellular vesicles (EVs), such as exosomes, that in fact contain distinct redox active molecules, are gaining more and more attention for their specific function in transcellular communication (Figure 2).

### Extracellular Redoxins

Extracellular Trx proteins have been found in different fluids of the body, including sputum, breast milk, umbilical cord blood, blood, cerebrospinal fluid and urine, and elevated levels have been correlated to various different pathologies [10] (see chapter 2). Most of the findings in the literature have analyzed the presence of Thioredoxin 1 (Trx1), which was originally identified as the released protein adult T cell leukemia-derived factor. The truncated, catalytically inactive form, named Trx80, is cleaved by the metallo protease ADAM17 [22]. Trx1 lacks a signal peptide and inhibition of the classical pathway did not influence Trx1 secretion. It was suggested that due to the small size of only 12 kDa, Trx1 might be able to cross the membrane directly or via unconventional secretory pathways. Recent data also indicated its presence in extracellular vesicles [12]. Even though it is still often referred to as antioxidant, the anti-inflammatory functions have been attributed to the regulation of specific thiol switches and pathways. Extracellular Trx1 is a chemotaxis suppressor. It suppresses, for instance, macrophage migration [23] and neutrophil migration by inhibiting their adhesion to endothelial cells, and it avoids complement activation [24]. Trx80, on the other side, is known to induce monocytes to secrete IL-12, stimulating a Th1 response [25]. Full-length Trx1 is able to cleave a disulfide bond in the ectodomains of TRPC5 [26], β1α7 integrin [27] and also tumor necrosis factor receptor superfamily member 8/CD30 [28]. In order for its enzymatic activity, Trx1 receives electrons from TrxR1 that was also shown to be present extracellularly. So far, the secretory mechanism for TrxR has not been extensively studied, however, it seems that it can be released by classical and alternative mechanisms [29]. Since TrxR is a NADPH-dependent enzyme, it is not known whether its enzymatic function can be recovered in the extracellular space. Interestingly, a study analyzed endothelial microvesicles to answer the question if MVs (i) contain large amounts of NADPH, (ii) are able to synthesize it or (iii) can uptake it from the extracellular space, e.g., plasma. The authors demonstrated that MVs indeed have the capacity to produce NADPH [30]. Moreover, NADPH is present in exosomes, as recently detected by lifetime imaging microscopy [31]. Thereby, exosomes contain a functional Trx system, i.e., NADPH, TrxR and Trx1, and, moreover, Prx1 and Prx2 (Figure 2).

Glutaredoxin 1 and 2 were also found extracellularly [32]. Similar to Trx1, they do not have a signal peptide and are not secreted via the classical pathway. It is likely that most of the proteins belonging to the Trx family proteins that lack a leader-peptide, are secreted via extracellular vesicles since a great number of redox active members of this family have been found in extracellular vesicles data bases [33,34]. In fact, we have found all four Grxs, i.e., Grx1, 2, 3 and 5, to be present in extracellular vesicles according to the two databases Exocarta [34] and Vesiclepedia [33]. These databases are compendia from proteins and RNA cargo from several nanoparticles, ranging from the most renowned exosomes and microvesicles to the sometimes-neglected apoptotic bodies and large dense core vesicles. Even though they state the fluid from where they have been identified and the used EV isolation method, the provided information only states the presence of the protein in an extracellular vesicle preparation, and therefore could sometimes also include the presence of proteins that have been described to be released via conventional pathways. Ongoing research in this field is important to understand these findings. For this reason, an initiative by many researchers in the EV field created a so-called knowledgebase (EV-TRACK [35]), which considers different quality controls in EV experiments, to try to ensure the transparency and replicability of results. For Grxs, no extracellular functions have been described, so far. With the presence of extracellular GSH, the reduction of oxidized glutaredoxins could generally be possible. In addition to the regulation of dithiol–disulfide exchange reactions, Grxs also catalyze de-/glutathionylation. Specific extracellular proteins were identified to be glutathionylated, such as albumin [36], transthyretin [37] and the redox proteins Thioredoxin and Peroxiredoxin [38]. Whether glutathionylation can actually take place in the extracellular space or not is unknown. It might depend on the extracellular body fluid and the levels of cysteine/cystine and GSH/GSSG. However, it is known that proteins can be released in their glutathionylated form, for instance, by LPS-stimulated macrophages [38]. The underlying mechanism is still elusive. It is, however, tempting to speculate that glutathionylation could act as a specific modification that marks proteins for cellular export, rather than protecting them from oxidation due to the inflammatory environment.

Peroxiredoxins are characterized by the Trx fold, peroxidase activity and distinct subcellular localization. They were long considered as antioxidants and unspecific peroxidases. They are, nowadays, rather considered as peroxide sensors that can transduce oxidation to substrate proteins and thereby mediate signal transduction. Prx1 and 2 have been shown to be released via exosomes. Interestingly, the secretion depends on oxidation. This involves mutating the catalytic cysteine residues, which are essential for their peroxidase function, inhibits Prx release [39]. Additionally, not only the presence of Prxs has been described in human EVs, but also the presence of their mRNAs has been found in the Evs’ lumen [33]. Prx1 and 2 were formerly described as NK-cell enhancing factors, however, the underlying regulatory mechanism was not elucidated [40]. Extracellular Prxs have been described as DAMPs and PAMPs, which specifically bind to TLR4 and are capable of activating TLR signaling and the NF-kB activation [41,42]. Studying the Exocarta and Vesiclepedia databases, we found all six isoforms to be released in extracellular vesicles. However, Prx4 is the only isoform that contains a signal peptide, and it was shown to follow the classical secretory pathway. The same is true for protein disulfide isomerases (PDIs), i.e., PDI A1, A3 and A6, which catalyze disulfide-isomerization reactions [11,12]. For instance, the function of PDIs has been linked to thrombosis, infection and inflammation, demonstrating that extracellular redox regulation is not limited to the regulation of single Cys modifications and disulfide reduction, but also the transfer of disulfides to target-soluble proteins or surface-exposed cellular structures. In fact, the metalloprotease ADAM17 is controlled via a PDI-mediated thiol switch [12]. Redox mediators, their release mechanisms and functions are summarized in Table 1.

## 2. Redox Regulation in and between Health and Disease

Redox regulation is essential for not only physiology and health, but also for the onset and progression of diseases. Various original articles and reviews address the role of ROS, reactive nitrogen species (RNS), antioxidants and/or redox proteins in ageing and different pathologies. However, many studies lack specificity. The terms ROS and RNS have been widely used, usually with a negative connotation, without considering the specific role of particular species in cell signaling. With increasing knowledge in redox biochemistry, life sciences and medicine, it is important to translate these findings and new perspectives to the medical practice. In fact, extracellular changes can be assessed in different body fluids of patients without using invasive and expensive techniques. By acknowledging extracellular redox regulation and monitoring pathological changes, we believe that we will be able to understand if something in our complex system is altered or slowly failing, for instance, in intermediate phenotypes of diseases, before a particular disease has progressed or manifested. It is acknowledged that the mutation or changes in a single element in a complex signaling pathway lead to the perturbation of the cell homeostasis, expressing as signs and symptoms that are shared between many diseases [53]. Focusing on solving the disease once signs and symptoms are already expressed has been the function of medicine in the last century, and most of the treatments have been directed on treating those signs and symptoms. However, multidisciplinary health teams have also tried to emphasize the importance of prevention [54]. We believe that specific redox changes could be established as biomarkers and tools for prevention, (early) diagnosis and prognosis. Some of the most prevalent diseases have common clusters in the “omics” (proteomics, metabolomics, genomics), explaining why different disorders have similar risk factors. Many of these proteomics, genomics and metabolomics patterns contain at least one redox-related molecule or protein [55], such as NADPH oxidases and their enzymatic products. As we mentioned before, the balance is an outdated model for oxidative stress. With that in mind, the clinical eye might look at altered levels of redox active molecules and proteins, and see them as a specific response to cope with, e.g., ischemia, inflammation or, generally, changes in the cellular microenvironment. These changes can be clinically relevant as biomarkers, risk factors and/or drug targets because they regulate critical cellular processes that may eventually lead to neoplasia or cell death. Here, we summarize studies that have assessed redox changes and thiol modifications in body fluids of healthy humans and in patients suffering from different diseases. Interestingly, some studies indicate potential correlations to the state of the disease.

### 2.1. Extracellular Redox Changes in Intermediate Pathophenotypes

Inflammation and immune response: Immune responses occur upon specific signals linked to pathogen-invasion or tissue damage in order to regulate pathogen-elimination or wound healing and limit tissue damage. However, inflammation can also persist in time, leading to adaptive changes in the affected tissues, as well as chronic immune cell infiltration. Chronic inflammation is associated with the most prevalent pathologies, such as heart diseases, diabetes, cancer, and autoimmune diseases. Redox regulation plays an essential role in both acute and chronic inflammation [56,57]. The involvement of redox active second messengers such as H_2_O_2_ and NO, redox proteins such as Prxs [58] and specific intra- and extracellular thiol switches of transducer and effector proteins can be acknowledged. The latter include, for instance, A disintegrin and metalloprotease 17 (ADAM17), High Mobility Group Box 1 (HMGB1), Myeloid differentiation primary response 88 (Myd88) and Nuclear Factor kappa B (NFκB).

The presence of extracellular redox proteins, particularly Trx1 and Prxs, and the oxidized proteins has been linked to different diseases related to infection and inflammation (reviewed in [57]). However, the activation of inflammation cannot fully explain the clinic of the diverse pathologies where for instance Prxs are elevated, bringing into consideration that the redox state of the redoxins is as crucial in understanding their extracellular function as their mere presence. The role of Trx family proteins in inflammation has been studied in sepsis, one of the leading causes of death in hospitalization areas. Redoxins in systemic inflammatory response syndrome (SIRS) are noticeably altered when compared to healthy controls. Interestingly, oxidation of erythrocyte Prx2 occurs during inflammation, e.g., by activated neutrophils [59]. Many proteins associated with redox regulation, such as myeloperoxidase, Prx3, and SOD2, increase in EVs in sepsis [60]. Even though the exact function of these proteins in the extracellular space is unknown, it shows that redox changes or “redox-messages” are delivered systemically via EVs upon inflammation.

The functional analysis of extracellular thiol switches is rare. The best studied example is the immune mediator and danger signal HMGB1 that contains three Cys residues and has different functions depending on its redox state. It is released via different pathways in its reduced or oxidized form. Zandarashvili and coworkers have shown that it is initially released in its reduced form via alternative secretion and is oxidized in the extracellular space, where its oxidation kinetics strongly depend on the microenvironment and ligand binding [61]. On the contrary, Kwak and colleagues have shown that disulfide formation is catalyzed by Prx1 and 2 in the nucleus and affects translocation to the cytoplasm and HMGB1 release [62]. HMGB1 oxidation is a regulated process during physiological inflammation and disease. Many different diseases have been linked to the levels of HMGB1 [63,64]. Interestingly, ongoing research ex vivo and in different mouse models links oxidized HMGB1 to angiogenesis in cancer [65], depression due to neuroinflammation [66] and sepsis, by increasing cell metabolism and the release of pro-inflammatory cytokines—two hallmarks of sepsis. In addition, blood GSH levels decrease with the severity of the inflammatory state. If GSH, which reduces HMGB1, decreases, then the pro-inflammatory effect of oxidized HMGB1 is maintained [67].

#### 2.1.1. Thrombosis

Hemostasis involves three main elements, blood vessels, platelets, and coagulation cascade components. As expected, these elements can also be redox-regulated. Decreased expression of endothelial TrxR2 has been associated with decreased flow-dependent vasodilation, which is linked to microthrombi formation [68]. Grx and Trx have been correlated with non-atherosclerotic blood vessels, both of them being expressed by cells in the intima, media, and adventitia, whereas their expression is decreased in the atherosclerotic lesion [69]. Mainly, PDIs have been long known for their role in thrombi formation. They are immediately secreted and have been shown to interact and regulate platelet factor V [70], factor XI [71] and tissue factor [72,73]. The latter is also regulated via glutathionylation, nitrosylation and by Trx1 [73]. PDI also regulate a physiological relevant thiol switch in the Von Willebrand factor (VWF), and their absence inhibits thrombus formation [74]. Moreover, the interaction of VWF with the plasma protein beta2GPI is regulated by extracellular Trx1 that reduces a specific disulfide bond. Reduced beta2GPI binds to VWF and increases platelet adhesion [75]. Interestingly, a recent study demonstrates that extracellular PDI becomes oxidized by Ero1a that in turn leads to the oxidation of plasma GSH creating a microenvironment that enables and promotes platelet aggregation [76].

#### 2.1.2. Fibrosis

Scar formation is a physiological repair mechanism after tissue damage. Fibrosis can occur as a possible outcome of inflammation and the thiol related family proteins, and second messengers are involved in this process. Fibrosis is characterized by the accumulation of matrix proteins such as collagen, and in physiological conditions, the repair is limited to the site of injury [77]. When fibrosis is not limited in space or time, organ function might be compromised. Numerous extracellular mediators are involved in scar formation. TGF-b is one of the central cytokines involved in scar formation and fibrosis [78], leading to increased NOX2 expression and superoxide and H_2_O_2_ production [79], suppression of Grx1 [80] and Gpx [81] and suppression of MAPK and AP-1 signaling [79]. Interestingly, at the same time, TGF-b can be redox-regulated itself. Oxidation activates TGF-b. Therefore, its activation could be connected to the microenvironment in chronic inflammation and fibrosis [82]. At the same time, TGF-b is also involved in maintaining an oxidizing environment due to the enzymatic production of reactive species and the decreased production of GSH, as seen in patients with pulmonary fibrosis and liver cirrhosis [43] and decreased expression of Grx1 in lung pathologies that progress to fibrosis [80]. H2O2 is also a key player in fibrosis, an effect that has been studied in the liver. Stellate cells in the liver could either become activated or undergo apoptosis depending on the amount of H2O2 generated by NOX proteins [83]. When activated, these cells increase the secretion of proteins into the extracellular space and fibrotic scar formation [83].

### 2.2. Extracellular Redox Changes in Pathologies

Among all the extracellular fluid compartments, the blood plasma (or intravascular compartment) is the most intensively studied. However, in addition to this, two other compartments exist extracellularly: the interstitial fluid and the so-called “transcellular” fluid. All these three extracellular fluids are regulated by tissue metabolism and are crucial in maintaining homeostasis, distributing nutrients, removing waste products, and for cell communication [84,85]. The “trapped” character of the transcellular water makes this extracellular fluid highly relevant for specific organ function such as the heart (pericardial fluid), the central nervous system (cerebrospinal fluid, CSF), eyes (vitreous humor), joints (synovial fluid), etc. The clinical and diagnostic utility of it is still being recognized and few studies have measured redox changes in those fluid compartments so far, with CSF and synovial fluids being the most studied. In this section, we discuss relevant changes of redox second messengers and redox and related proteins in the extracellular space observed in common human pathologies.

#### 2.2.1. Cardiovascular System

It is well known that Trx1 levels increase in the plasma and serum of patients after myocardial infarction [86,87]. However, substantial elevation of plasma/serum Trx1 appears to be restricted to the first hours/days after an acute injury to the heart. For instance, acute myocardial infarction patients showed higher levels of Trx1 on admission than patients with stable exertional angina, but declined 12 h thereafter [86]. Similarly, serum Trx1 levels of MI patients with ST segment elevation correlated with common early markers of cardiac tissue damage such as initial creatine kinase, cardiac-specific troponin, and peak creatine kinase [88]. In non-survivor cardiac arrest patients, plasma Trx1 levels were significantly higher on admission and day 1 compared to those of survivors [87]. This is not necessarily bad, as Trx1 can serve as a predictor for myocardial damage and disease outcome in humans. In more chronic disease processes, even though Trx1 elevation in plasma is less substantial, it can still be useful for monitoring disease progression. One example is chronic heart failure. In those patients, plasma Trx1 levels were clearly lower than those observed in acute myocardial infarction (32 ng/mL vs. 103 ng/mL, respectively), but even so, they were still significantly higher than in healthy subjects and increased even more with advanced stages of the disease [86,89]. Jekell et al. also analyzed other blood parameters and found a strong correlation with lipid peroxides in the blood and common markers associated with chronic cardiac failure such as circulatory P-selectin, serum creatinine, as well as free salivary cortisol [89]. This makes extracellular Trx1 also an interesting blood component to monitor disease progression and general welfare of the patients. Trx1 is also significantly elevated in the plasma of patients with peripheral arterial disease 4 h after angioplasty [90] and Trx1 and Prx1 have been described in the pathogenesis and onset of atherosclerosis, a first step in the development of ischemic heart disease.

Assuming extracellular Trx1 is actively secreted by cells suffering from dysregulated redox signaling, the source could be the primarily affected tissue or a remote tissue as recently published [91]. Extracellular Trx1 can likely be found in three different forms: (1) full-length soluble Trx1, (2) truncated Trx-80, and (3) Trx1 inside extracellular vesicles. As previously noted, full-length extracellular Trx1 plays a role in the regulation of immune cells function [92], in the reduction of extracellular thiol protein groups [93], and as an electron donor to the human plasma peroxidase [94]. The small molecular weight of Trx1, however, makes it highly filterable at the glomeruli and thus its half-life is about 1 h. Fusion of Trx1 to human plasma albumin extended its half-life ten times and has proven to be beneficial in a mouse model of ovalbumin-induced lung injury [95]. The reductase activity of Trx1 also relies on the presence of TrxR1 and NADPH. TrxR1 was long ago reported to be secreted from cells into the blood plasma [29]. The extracellular source of NADPH, however, has not been clarified. Only recently, NADPH has been detected in extracellular vesicles using lifetime imaging microscopy [31]. Thus, extracellular vesicles would be equipped with a full Trx system that is capable of sustaining Trx1’s oxidoreductase activity. Interestingly, truncated and redox inactive Trx80 is also elevated in the plasma during inflammatory processes and Couchie et al. found this protein to be significantly increased in aged patients, whereas plasma Trx1 levels dropped in the same group [52]. This can be attributed to cleavage of Trx1 in old patients, since ADAM-10 and ADAM-17 expressions were significantly higher in PBMCs of the same patients. Moreover, Trx80 showed a high pro-inflammatory and pro-atherosclerotic profile in in vitro and in vivo experimental settings, which could be a sign of the generalized inflammatory predisposition in aging patients. Several aspects regarding the role of extracellular redoxins in cardiovascular disease need to be elucidated, among others, the source cells that actively release these proteins into the extracellular compartments. The transcellular fluid relevant to cardiovascular system’s function, specifically the heart, is the pericardial fluid. During cardiac surgery, the pericardium is damaged, which induces infiltration of neutrophils and monocytes and subsequently, the formation of O_2_^−^ and H_2_O_2_ during the respiratory burst [96]. Kramer et al. also analyzed the pericardial fluid of patients 4 to 48 h after coronary bypass surgery, valve replacement, or valve repair, and identified evidence of protein thiol oxidation and protein carbonylation, as well as free hemoglobin and methemoglobin. It would be very interesting to analyze the levels of oxidoreductases in this fluid in the future.

#### 2.2.2. Respiratory System

The airways are continuously exposed to potentially harmful airborne particles and pollutants. As a result of the interaction of these environmental substances and gases with the respiratory epithelium, reactive oxygen species are generated [97]. ROS and the inflammatory response cause significant damage to the airways. Extracellular Trx1 has recently been shown to be beneficial under experimental exposure to urban pollutants in mice. Intravenous administration of human serum albumin-fused Trx1 suppressed the damage induced by urban aerosols in mice [98]. Classically, redox proteins have been investigated in lung diseases such as acute lung injury, asthma, and chronic obstructive pulmonary disease (COPD) which are associated with disrupted redox signaling and regulation [97]. Early studies on redox proteins in patients suffering from these diseases reported elevations of Trx1 levels in serum or plasma. For instance, Trx1 was elevated in the serum of patients with acute exacerbation of asthma, however, no differences were observed in terms of sex, smoking, or corticosteroid treatment [99]. Smoking is associated with more oxidized redox couples in the plasma such as GSH/GSSG and Cys/CySS [100]. Under these oxidative extracellular conditions, Grx1 may become relevant as a key reductase involved in the regulation of S-glutathionylated proteins. Indeed Grx1 was found elevated in the sputum both in the cells and supernatants of acute exacerbated COPD patients [101]. Recently, Grx1 function was also associated with the regulation of airway fibrosis in a mouse model [102]. In the same study, Grx1 knockout mice showed enhanced collagen deposition after exposure to house dust mite as well as increased levels of transforming growth factor beta 1 (TGFB1) in the bronchoalveolar lavage which controls the proliferation and differentiation of basal cells in the alveolar epithelium. In a study of sputa by Rostilla et al. [103], extracellular Trx1 levels had a negative correlation with asbestos exposure, yet a positive correlation with smoking and lung cancer. A similar increase of Prx2 was detected in the sputa of smokers and lung cancer patients. Additionally, Grx1 was elevated in sputum samples of asthmatic patients [104]. Recently, proteomic analysis of bronchoalveolar lavage fluid obtained from cystic fibrosis patients revealed higher levels of SOD2 and Gpx3 in respiratory exosomes. Antitumoral treatments are in some cases associated with lung damage, as seen in patients receiving gefitinib who develop interstitial lung disease. In those patients, serum Trx1 levels are significantly increased during the duration of the therapy but decrease after cessation of the therapy [105]. Thus, extracellular redox proteins may represent, also in the lungs, interesting targets for therapy and diagnostic purposes. Indeed, many efforts have been made in order to characterize Grx1 function in lung diseases and studies using recombinant Grx1 have shown promising results. As GSH levels are altered in pulmonary diseases such as idiopathic pulmonary fibrosis and that Grx1 has the ability to reduce S-glutathionylated proteins, studies using recombinant Grx1 administration have gained a special attention in the redox field. Anathy et al. conducted an excellent study using samples from idiopathic pulmonary fibrosis patients, transgenic and Grx1 knockout mice, as well as recombinant Grx1 treatments applied directly into the airways of fibrotic mice [106]. The authors found that lung fibrosis is associated with increased protein-S-glutathionylation and apoptosis, as well as decreased Grx1 levels and activity, and that recombinant Grx1 administration increased Grx1 activity and decreased fibrosis in a mouse model. All these observations allow a very optimistic outlook towards the future development of extracellular redox proteins as novel therapeutic and diagnostic tools for respiratory diseases.

#### 2.2.3. Neurological Disorders

Neurodegenerative and neuroinflammatory diseases are clusters in neurology and are associated with redox changes and changes in ROS and RNS production and removal. In early stages of Alzheimer’s disease (AD), increased expression of human beta defensins (hBD) has been described [107]. Notably, an unbalance in the expression of the constitutively hBD-1 and the inducible hBD-2 indicates a possible connection with the inflammatory phenotype in neurodegenerative disease [108]. Interestingly, hBD-1 is known to be reduced by Trx and Grx [107], although a reduced molecule is necessary to form bacteria-entrapping nets, the role of hBD in neurodegeneration, where their predominant feature is protein aggregation, has not been entirely assessed. In the nervous tissue, extracellular redox protein revealed themselves once again as potential markers of disease. The CSF represents the transcellular fluid of relevance in the central nervous system and both Trx1 and Grx1 are increased in the CSF of early-stage AD patients and correlated with standard AD biomarkers such as tau and phosphor-tau [109].

Multiple sclerosis (MS) is one of the most frequent diseases affecting the nervous system of young adults and it has also been associated with dysregulated redox signaling [110,111]. In MS patients, several redox-induced changes in blood serum proteins were reported, such as increased urinary 8-iso-PGF2α [112], higher thiobarbituric acid reacting substances and advanced oxidation protein products [113]. Extracellular redox proteins are also altered in multiple sclerosis patients. Serum Trx1 levels were increased in newly diagnosed MS patients compared to control and to MS patients receiving immunotherapies [114], but declined in relapsing-remitting MS patients without treatment compared to healthy controls or to patients under ongoing treatment [115]. These results show, once again, the potential value of extracellular redox protein changes in monitoring degenerative disease progression.

Extracellular peroxiredoxins have attracted the attention of several research groups in the field of stroke and brain injury both from a diagnostic, as well as from an immunological point of view. Peroxiredoxin 6 was shown to be increased in the plasma of patients diagnosed with traumatic brain injury, for as long as seven days post insult [116], which can be explored as a nervous tissue damage marker. In addition to this, stroke and subarachnoid hemorrhage models have revealed a key role of extracellular peroxiredoxins in activating the innate immunity via Toll-like receptors 2 and 4, as novel damage-associated molecular pattern molecules (DAMPs) in the CNS [117,118]. Thus, extracellular redox research may lead to novel diagnostic and therapeutic approaches in nervous tissue diseases.

#### 2.2.4. Autoimmune Diseases

Autoimmunity is the lack of tolerance towards self-antigens. Where and when this tolerance is broken is an ongoing research topic. Protein modification is one of the hypotheses of autoimmunity; those peptide modifications can be the consequence of redox reactions. Examples of oxidation of proteins are seen in systemic lupus erythematosus (SLE), where auto-antibodies against oxidized low-density lipoprotein (oxLDL) are identified and are associated with thrombosis in those patients [119]. Additionally, in type 1 diabetes (T1D), the presence of a disulfide bond on the A-chain of insulin enables the recognition by HLADR4+ CD4+ T-cells clones in patients, but not in healthy subjects [120]. Rheumatoid arthritis (RA) is another autoimmune disease where redox changes have been observed [121]. An early study reported increased levels of Trx1 in the synovial fluid and the synovial tissue of RA patients compared with patients with other joint diseases such as osteoarthritis and gout [122]. In addition to Trx1 synovial fluid increase, also plasma Trx1 levels are reported to increase significantly in RA patients and correlated with disease activity and serum C reactive protein levels [123].

#### 2.2.5. Cancer

The extracellular space is gaining increasing recognition as a key compartment determining cancer cell survival and evolution [124,125]. Despite this, redox proteins’ role in cancer cells has been mostly investigated intracellularly and less attention has been paid to extracellular redox changes. Redox protein changes may reveal novel applications of these proteins in the development of novel diagnostic markers and therapeutics. Trx1, for instance, can serve as a biomarker in hepatocellular carcinoma, as it is elevated in early stages of the disease [126]. In addition to this, Trx1 plasma levels were also significantly higher in patients with pancreatic carcinoma as compared with healthy control [127]. In gastric cancer, adding TrxR to the screening increases the assessment of therapeutic efficiency, whereas TrxR is also increased in this neoplasia [128]. Non-small cell lung cancer (NSCLC) is associated with increased plasma levels of thioredoxin reductase and higher expression of this protein in the tumor, which in this case does not only serve as a biomarker, but also as a possible differential diagnostic criterion in lung cancer [129]. Moreover, in NSCLC, Prx6 can be potentially used as a marker for monitoring the efficiency of anti-epidermal growth factor receptor therapy [130]. From a therapeutic point of view, the extracellular redox state represents an interesting area of development, as it is considered a key regulator of cell proliferation and angiogenesis, two essential conditions for tumor growth. Accordingly, increased extracellular levels of ROS, such as O_2_^−^, resulted in an increase in cancer cell invasion ability [131]. On the other side, overexpression of extracellular SOD [131] and GPx3 [132] resulted in inhibition of cancer cells invasion ability and growth. Moreover, extracellular redox changes induced by the simultaneous overexpression of extracellular SOD and the knockdown of xCT showed promising results in inhibiting prostate cancer cell invasion [133].

## 3. Redoxins and Thiols as Biomarkers, Risk Factors and/or Drug Targets

Understanding the function of extracellular regulation is important to understand human health and the onset and progression of diseases. Even though standardized range values for many redox active molecules are still not available, their measurement in different body fluids and extracellular compartments could provide us with valuable information on healthy organ systems, and if something is altered or slowly failing. Establishing the precise functions of redox proteins, low-molecular-weight thiols and reactive species and their impact in the development of pathologies requires the ability to (1) measure them in biofluids, (2) quantify the redox state of their substrates and determine the potential irreversible oxidative damage that accumulates.

### 3.1. Reactive Oxygen and Nitrogen Species

Direct identification of ROS and RNS in vivo is a challenge, due to the short half-life most radicals and reactive species have. They react rapidly with redox enzymes or molecules in their vicinity. In addition, ROS and RNS are produced as part of cell signaling and as such during an immune response. Therefore, measuring the levels of reactive species must be individualized to the context and correlated to other factors. In the case of Chronic Granulomatosis Disease (CGD), where phagocytes express a defective NOX, the lower superoxide production seems to be associated with the severity of the disease. The measurement of the production of reactive species could help with patient prognosis [134]. ROS measurements by luminometry have been shown to have diagnostic or prognostic value in different body fluids, such as in the CSF of patients with meningitis [135], as well as with idiopathic tinnitus where an increase of ROS in the serum has been described [136]. Moreover, the isolation of PBMCs or specific sub populations of immune cells and the subsequent analysis of ROS, GSH and/or the gene expression or activities of antioxidants and redox active proteins has been studied in different diseases. For instance, CD4 positive T cells isolated from patients suffering from RRMS show reduced expression of catalase and increased expression of Nrf2 and SOD1 [137] and reduced activities of SOD and Gpx [138]. Interestingly, higher levels of ROS and O_2_^•−^ were detected in the relapse compared to remission phase [137]. Some therapeutical strategies focus on the use or induction of reactive species. Medical gas therapies have been used in different clinical fields. NO, for instance, is used for pulmonary diseases, in lung failure in premature neonates and in ischemia [139,140,141]; H_2_S for vascular diseases [142]. Induction of reactive oxygen and nitrogen species (including ozone (O_3_), O_2_^•−^, hydroxyl radical (^•^OH), H_2_O_2_, NO, peroxynitrite (ONOO^−^)) by cold physical plasma has antimicrobial and wound healing promoting activity. It is used in dermatology to treat wounds and infections. It is being tested for the potential of biofilm removal and in different oncological clinical studies (reviewed in [143]). ROS-induction treatments play an important role in cancer therapy in the forms of chemotherapeutic drugs, photodynamic therapy, radiotherapy, as well as during immune therapy (reviewed in [144]).

### 3.2. Oxidation Products

Many markers have been developed to quantify oxidative distress in biological samples. In the case of nucleic acid oxidation, 8-hydroxy-2′deoxyguanosine (8OHodG), 5-chlorocytosine, 5-chlorouracil, εdA, εdC, have been described [145]. Marrocco and coworkers reviewed the oxidation of nucleic acids, the relationship with high incidence pathologies and the technical advantages and limitations [146]. Some products of lipid peroxidation are HNE, MDA, F2-lsop, MPTP, TBARS. For example, an increase in HNE (4-hydroxy 2-nonenal) has been associated with activation of NF-kB and MAPK signaling pathways in neurodegenerative diseases, multiple sclerosis, cancer, and atherosclerosis [147]. Measuring reduced and oxidized GSH and the GSH:GSSG ratio has also been a marker for oxidative distress.

Advances in sensitive and cost-efficient techniques to detect and quantify proteins and oxidation products in biofluids contribute to their applications in clinical routine [148]. Specific posttranslational modifications of proteins in human studies and potentially as a diagnostic method are technically evolving [149]. In the case of oxidation, a few clinically relevant oxidized proteins are known: One important protein in which redox modification has been associated with poor prognosis in several diseases is albumin [150,151]. When ROS increases in the circulation, redox changes in the metal and fatty acid-binding sites of albumin decrease, increasing the oxidative potential of these free molecules. Interestingly, different forms of oxidized albumin have been described [36], which were linked to diseases including diabetes mellitus [152] and liver cirrhosis [153]. Interestingly, the levels of oxidized albumin correlated with progression of liver cirrhosis [154]. Tanaka and colleagues analyzed the presence of different redox states of albumin over 180 days, following kidney transplantation demonstrating the temporal heterogeneity [155]. Another example of heterogeneity is the transport protein transthyretin that contains Cys10 which can be S-cysteinylated, S-cysteinglycinylated and S-glutathionylated [156]. In vitro analyses showed different amylogenic properties of the differently modified proteins [157]. This was also shown in vitro for Cofilin-1 that exists in different redox states [158]. In a global approach to study oxidative modified serum proteins in patients suffering from relapsing-remitting multiple sclerosis (RR-MS), oxidation of vitamin D-binding protein and apolipoprotein A-IV increased from remission to relapse [159]. Another oxidized protein that has been described in neuroinflammation and manifested pathologies is HMGB1, i.e., depression due to neuroinflammation [66], angiogenesis in cancer [65] and sepsis [67]. Interestingly, reduced and the disulfide-form of HMGB1 are targets for salicylic acid [160]. Stable and highly abundant protein oxidation products constitute promising biomarker candidates such as pentraxin 3 in sepsis [161], methionine oxidation of albumin [162,163] and oxidation of hemoglobin [164,165], oxidation of low-density lipoprotein [166,167]. Vitamin D-binding protein and Apolipoprotein A-IV oxidation have been associated with MS relapses [159].

### 3.3. Antioxidants and Redox Enzymes

Antioxidants such as the low-molecular-weight thiol GSH have been analyzed and quantified in various body fluids and pathologies. A decrease in GSH in epithelial lining fluid (ELF) has been found in patients suffering from idiopathic pulmonary fibrosis (IPF) [168]. As a strategy to augment GSH levels, an aerosol treatment of glutathione or intravenous administration of the GSH-precursor N-acetyl cysteine (NAC) showed promising results and restored GSH levels [169,170]. A different study tested an oral NAC therapy, which led to increased levels of GSH in the bronchoalveolar lavage fluid, however, not in ELF [171]. A decrease of GSH was also found in seropositive HIV patients in ELF [172] and plasma [173]. Interestingly, the changes in ELF were not detected in patients of an early disease stage [174]. NAC treatment has been linked to lower oxidative stress and less inflammation. NAC has been studied in clinical trials as a treatment or adjuvant therapy in several conditions including pneumonia [175], chemotherapy-induced oral mucositis [176] and chronic obstructive pulmonary disease (COPD) [177]. In addition, NAC administration affects viral infections caused, e.g., by influenza [178] or COVID-19 [179,180]. NAC has been known for a long time as a powerful antioxidant and as an antidote for paracetamol overdose [181]. It also shows beneficial outcomes after surgery. It was shown to decrease the incidence of acute kidney injury (AKI) after heart surgery [182] and prevented changes related to oxidative stress in routine arthroscopic knee surgery [183]. NAC ingestion followed by hyperoxia showed increased levels of erythropoietin, an effect that was not seen in the group only exposed to hyperoxia [184]. This could be a potential adjuvant treatment in some anemias. Another promising preventive and therapeutic agent are the Gpx mimic Ebselen that has antioxidant properties and is being tested in different clinical trials [185]. There are also different types of inhibitors against ROS-inducing enzymes, including NOX and NOS (reviewed in [186]). Many therapeutical drugs, such as aspirin, and natural compounds are known to modulate Nrf-2 activity (reviewed in [187]). The targeted pharmacological Nrf-2 activation is an approach to treat, for instance, cardiovascular or neurodegenerative diseases [188]. One potential first-line treatment for RRMS is dimethyl fumarate, which functions via the activation of the Nrf-2 pathway [189]. Nrf-2 inhibition, on the other hand, is an approach in cancer therapy [188].

Redox enzymes of the Trx family have also been analyzed in different pathologies. In hydronephrosis in the pediatric population, Trx is increased in plasma and urine and is associated with the severe clinic, interestingly, the ratio between these measurements gave more information regarding the clinical outcome [190]. Trx1 is elevated in distinct pathologies with the potential as biomarker, i.e., in acute coronary syndrome (ACS) and dilated cardiomyopathy, acute phase of myocarditis, unstable angina, and myocardial infarction [191], cardiac arrest [87], sepsis [192,193,194] rheumatoid arthritis [123] and stroke [195]. In stroke, Trx1 could also be used as a prognostic marker [196,197]. Both Trx1 and TrxR1 have been described as biomarker candidates in cancer: Trx1 in hepatocellular carcinoma [126] and breast cancer [198]; TrxR in gastric cancer (for diagnosis and therapeutic efficiency) [128], non-small cell lung cancer [129] and liver cancer [199]. Clinically, TrxR can be targeted and inhibited by auranofin, a drug used against rheumatoid arthritis (RA), anticancer and antiparasitic treatments [200]. In a multicenter randomized study, auranofin showed beneficial effect on clinical synovitis in patients with RA, as well as improvements in quality of life in these patients [201].

Inhibition of other members of the Trx family, PDIs, are being evaluated as a novel antithrombotic strategy [202]. Interestingly, Prx4 levels in serum can be associated with a mortality risk for cardiovascular disease [203]. Cerebrovascular disease is another prominent cause of morbidity and mortality in the world, time of onset is always a risk factor to consider, Prx1 could be integrated into the initial assessment of patients to determine the onset of the ischemic process [204], with the goal of saving more brain parenchyma. Prx1 has also been identified as a potential biomarker in EVs isolated from patients suffering from endometriosis [205]. Prx2, on the other hand, can be integrated as a quality control marker of RBCs stored for blood transfusions. The viability of RBCs from a donor needs to be assessed before transfusion. Unfortunately, blood banks cannot always ensure fresh blood to all patients. Therefore, Prx2 can be used to assess how healthy RBCs are [206]. More recently, increased levels of Prxs have been associated with decrease glomerular filtration rate in several chronic kidney diseases, such as IgA and membranous nephropathy, as well as lupus nephritis [207].

A crucial tool of possible use to the physicians in the clinic is the presence of autoantibodies against thioredoxin related proteins, where their presence has been associated with the outcome in different pathologies. In breast cancer, the presence of Thioredoxin like-2 autoantibodies was higher in triple-negative breast cancer samples, compared with other samples, estsblishing a connection with the sensibility to treatment. Prx autoantibodies have been associated with recurrent pregnancy loss, preeclampsia, increase neurological clinic manifestation in lupus, and associated with deep vein thrombosis [208,209,210].

## 4. Conclusions and Future Perspectives

Redox regulation has long been underestimated. Reactive species have exclusively been regarded as factors nonspecifically contributing to aging and progression of diseases, whereas redox proteins were seen as their counter players in the form of antioxidants. Even though decades of research have increased our knowledge on redox regulation of physiological processes, and the term oxidative stress has been redefined to indicate redox signaling, the former paradigm is deeply rooted in medical research.

**Figure 3:**
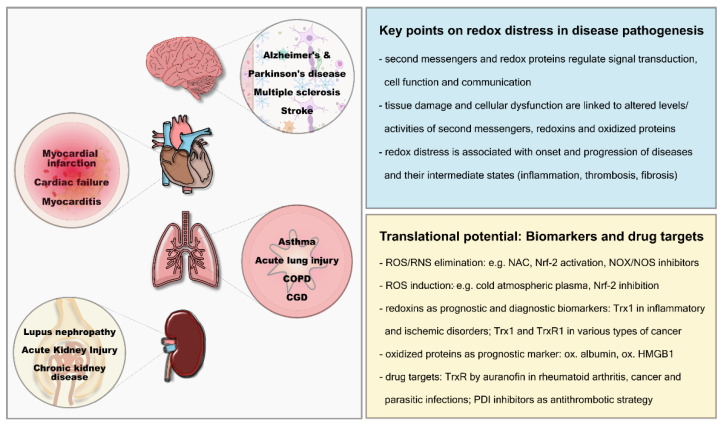
Redox regulation in translational medicine: Redox regulation is essential for the normal function of organs such as the brain, the heart, the lungs, and the kidneys. Alterations can lead to oxidative distress and the onset and progression of diseases. Extracellular changes can be analyzed in body fluids, these changes can be assessed as risk factors and have the potential to be utilized as prognostic and diagnostic biomarkers. New therapeutic strategies for different disorders include the induction or inhibition of specific reactive species, for instance, via Nrf-2 modulation, or the inhibition of specific enzymes such as TrxR and PDIs.

It is essential to understand the functional and clinical implications of dysregulated redox circuits for health and the onset and progression of disorders, also addressing earliest stages of diseases. This will help to build the bridge between mechanistic findings and pathophysiological implications on the bench to system-based translational approaches in the clinics (Figure 3). Particularly, extracellular changes can be analyzed in body fluids using minor or noninvasive techniques. These changes cannot only help to identify new and early biomarkers for prognostics and diagnostics, but, moreover, can lead to the establishment of innovative therapeutical strategies that target specific enzymes or thiol switches (Figure 3).

## Figures and Tables

**Table 1:** Extracellular redox mediators.

Redox Mediator	Release Mechanism	Function	Reference/(Vesiclepedia ID)
Second messengers			
H_2_O_2_	It crosses plasma membrane directly or via aquaporins	Signal transduction	[43]
H_2_S	It crosses plasma membrane directly	Signal transduction	[44]
NO	It crosses plasma membrane directly	Signal transduction, vasodilation	[45]
Low molecular weight redox couples			
Cysteine/cystine	Carrier-mediated: CPx		[46]
GSH/GSSG	Carrier-mediated: MRPs		
Redox enzymes			
Gpx3	Alternative release in extracellular vesicles	It catalyzes the reduction of H_2_O_2_ and lipid hydroperoxides	[47] [33] (VP_2878)
Gpx7, 8	Alternative release in extracellular vesicles	unknown	[33] (VP_493869, VP_2882)
Grx1, 2, 3, and 5	Alternative release in extracellular vesicles	unknown	[48] [33] (VP_2745, VP_51022, VP_10539, VP_51218)
PDI A1, A3, and A6	Classical release	Disulfide isomerisation	[12]
Prx1, 2, 3, 5, and 6	Alternative release in extracellular vesicles and exosomes	TLR-mediated signaling	[48] [33] (VP_5052, VP_7001, VP_10935, VP_9588, VP_9588
Prx4	Classical release	TLR4-mediated signaling	[49]
SOD3	Classical release	Catalysis of the dismutation of superoxide anion to H_2_O_2_	[50]
Trx1	Alternative release in extracellular vesicles and exosomes	Regulation of extracellular thiol switches, i.e., disulfide reduction	[48] [33] (VP_7295) [51]
Trx80	Alternative secretion, processed and released via ADAM17	Chemokine-like functions, no redox activity	[52]
TrxR1	Classical release and alternative release in extracellular vesicles	Unknown. Potential reduction of Trx1	[29] [33] (VP_7296)
